# Supplementary material for: Association of Vitamin D Supplementation with Glutathione Peroxidase (GPx) Activity, Interleukine-6 (IL-6) Levels, and Anxiety and Depression Scores in Patients with Post-COVID-19 Condition
Source: Int J Mol Sci. 2025 May 10;26(10):4582. doi: 10.3390/ijms26104582 (PMC12110956; doi:10.3390/ijms26104582)
Supplement: Supplementary file 1 [file ijms-26-04582-s001.zip › ijms-3580683- supplementary.pdf]

Table S1. Comparative analysis between Bolus vs Daily supplementation of the serum determination of GPx activity and IL-6 levels.

Type of supplementation			
**GPx (U/ mg protein)**	**Bolus**	**Daily**	p Value
Pre-treatment	145.09±40.74	212.64±88.77	0.006*
Post-treatment	205.07±119.47	257.64±114.26	0.57[&a]
**IL-6 (pg/mL)**	**Bolus**	**Daily**	p Value
Pre-treatment	6.97±12.05	3.02±6.62	0.73
Post-treatment	3.08±6.44	2.25±6.37	0.45

*Statistical significance p≤ 0.05, SD=Standard deviation

[&]The covariates that appear in the model were evaluated with the following value: GPx activity = 182.42, [a]Bonferroni Post Hoc Test

Table S2. Comparative analysis between Bolus vs Daily of the HADS Anxiety Score and HADS Depression Score

Type of supplementation			
**HADS Anxiety Score**	**Bolus**	**Daily**	p Value
Baseline	3.25±3.20	4.88±4.59	0.13
Post-treatment	1.74±2.64	0.35±0.97	0.01*
**HADS Depression Score**			
Baseline	2.86±3.30	4.85±5.46	0.10
Post-treatment	1.68±2.65	0.77±1.92	0.15

*Statistical significance p≤ 0.05, SD=Standard deviation

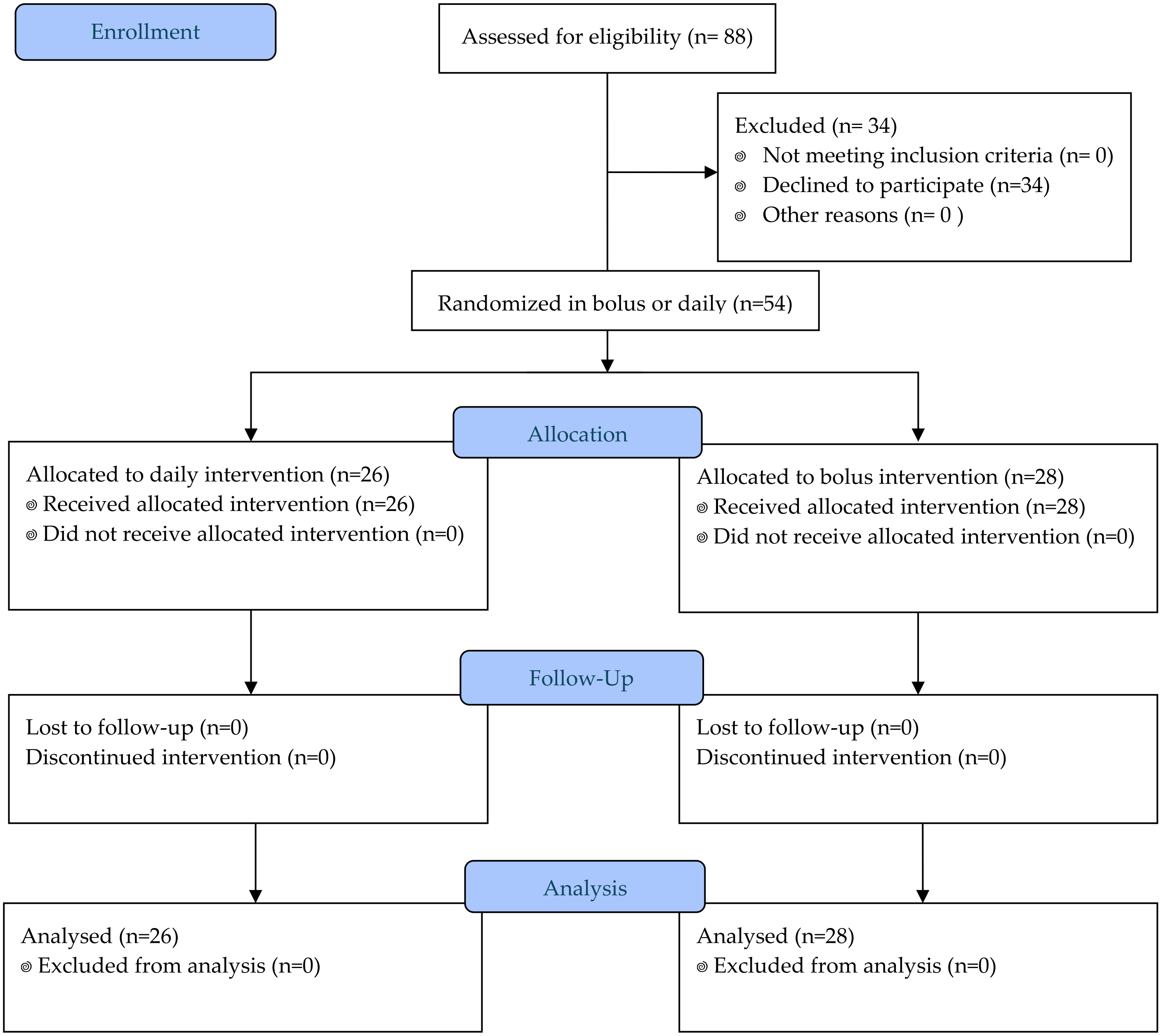

Figure S1. Consort Flow Diagram.
